# Circadian rhythm disturbance and delirium in ICU patients: a prospective cohort study

**DOI:** 10.1186/s12871-023-02163-4

**Published:** 2023-06-13

**Authors:** Jingjing Li, Shining Cai, Xiao Liu, Jinghua Mei, Wenyan Pan, Ming Zhong, Yuxia Zhang

**Affiliations:** 1 Department of Nursing, Zhongshan Hospital, Fudan University, Shanghai, China; 2 Department of Critical Care Medicine, Zhongshan Hospital, Fudan University, Shanghai, China; 3 School of Nursing, Fudan University, Shanghai, China

**Keywords:** Delirium, Circadian rhythm, Melatonin, Cortisol

## Abstract

**Background:**

Patients treated in the intensive care unit (ICU) may experience a reversal of day and night. The circadian rhythm in ICU patients can be disturbed.

**Methods:**

To explore the relationship between ICU delirium and the circadian rhythms of melatonin, cortisol and sleep. A prospective cohort study was carried out in a surgical ICU of a tertiary teaching hospital. Patients who were conscious during the ICU stay after surgery and were scheduled to stay in the ICU for more than 24 h were enrolled. Serum melatonin and plasma cortisol levels were measured three times a day by drawing arterial blood on the first three days after ICU admission. Daily sleep quality was assessed by the Richard-Campbell Sleep Questionnaire (RCSQ). The Confusion Assessment Method for the Intensive Care Unit (CAM-ICU) was performed twice a day to screen for ICU delirium.

**Results:**

A total of 76 patients were included in this study, and 17 patients developed delirium during their ICU stay. Melatonin levels were different at 8:00 (*p* = 0.048) on day 1, at 3:00 (*p* = 0.002) and at 8:00 (*p* = 0.009) on day 2, and at all three time points on day 3 (*p* = 0.032, 0.014, 0.047) between delirium and non-delirium patients. The plasma cortisol level in the delirium patients was significantly lower than that in the non-delirium patients at 16:00 on day 1 (*p* = 0.025). The changes in melatonin and cortisol secretion levels exhibited obvious biological rhythmicity in non-delirium patients (*p* < 0.001 for melatonin, *p* = 0.026 for cortisol), while no rhythmicity was found in melatonin and cortisol secretion levels in the delirium group (*p* = 0.064 for melatonin, *p* = 0.454 for cortisol). There was no significant difference in RCSQ scores in the first three days between the two groups.

**Conclusions:**

The disturbance of the circadian rhythm of melatonin and cortisol secretion was associated with the development of delirium in ICU patients. Clinical staff should pay more attention to the importance of maintaining patients’ normal circadian rhythms in the ICU.

**Trial registration:**

The study was registered with the US National Institutes of Health ClinicalTrials.gov(NCT05342987) (25/04/2022).

## Introduction

Delirium is a common complication occurring in patients who are admitted to intensive care units (ICUs), especially after major surgeries. The reported incidence of delirium in ICU patients varies from 11–87% [1–3]. Delirium causes a series of adverse consequences, including increased mortality [4, 5], length of ICU stay [6, 7] and medical costs [8]. Additionally, delirium has been proven to be independently associated with long-term cognitive impairment, which threatens the quality of life in ICU survivors [9].

Although there are several hypotheses to explain the mechanism of delirium, the pathogenesis of delirium is still not fully understood. Circadian rhythm disruptions have recently drawn increased attention as a possible mechanism underlying delirium [10]. This hypothesis suggested that disruptions to the 24-h circadian cycle, unusual stages of sleep and variations in natural light exposure may lead to disturbances in the physiological sleep architecture that may contribute to the development of delirium [11]. As circadian rhythm cannot be directly measured, the sleep–wake cycles of melatonin and cortisol secretion are commonly used as substitutes to assess circadian rhythmicity [6].

However, because of special environmental and treatment factors in the ICU, patients are more likely to have circadian rhythm disturbance in the ICU. A randomized controlled trial [12] performed in Denmark using polysomnography (PSG) to assess sleep quality revealed that 47% of ICU patients showed pathologic sleep patterns. Akpinar et al. [13] used the Richard-Campbell Sleep Questionnaire (RCSQ) to assess subjective sleep quality and found that the average level of sleep quality in ICU patients was low, and patients with worse sleep quality had an increased risk of delirium. In previous studies, the specific circadian rhythm in ICU patients and its relationship with the development of delirium were reported. However, the results are controversial. Dessap et al. [14] analysed melatonin levels using the 24-h excretion of melatonin’s urinary metabolite 6-sulfatoxymelatonin (aMT6s) and found that delirium was associated with an alteration in the circadian rhythm of melatonin excretion. Song et al. [15] found that delirium patients had a delayed cortisol rhythm compared with non-delirium patients, but the rhythm of melatonin was similar between the two groups.

Therefore, the present study aimed to investigate the circadian rhythm in ICU patients and explore the relationship between circadian rhythm disturbance and delirium.

## Materials and methods

### Design

This was a single-centre, prospective cohort study conducted at a tertiary teaching hospital. The study has been reported according to the Strengthening the Reporting of Observational Studies in Epidemiology (STROBE) statement [16]. The study was registered with the US National Institutes of Health ClinicalTrials.gov(NCT05342987) (25/04/2022).

### Participants

We consecutively recruited patients who were admitted to the surgical ICU from February 2022 to July 2022. The inclusion criteria were age > 18, Richmond Agitation-Sedation Scale (RASS) score > -4 (patient could be aroused by voice stimulation) and expected ICU stay longer than 24 h. The exclusion criteria were as follows: (a) history of mental or psychological illness; (b) remained in coma or deep sedation; (c) delirium occurring at the time of admission; (d) unable to fully participate in delirium testing, including blind, deaf, illiterate or unable to understand Chinese; and (e) neurosurgery and maternal patients.

### Data collection

The baseline demographic data and medical history were recorded by doctors and trained nurses. The Acute Physiology and Chronic Health Evaluation (APACHE) II score [17] was used to assess the severity of critically ill patients.

### Confounding control

The study was conducted in a surgical ICU, which is a windowless and closed ICU. We used a standardized lighting and noise control protocol in our ICU. Regularly, the artificial light is on during the daytime and off from 21:00 pm to 6:00 am, and the lights in the nursing station area remain on for 24 h. The lights above patients are turned on when interventions are needed during the nights. Melatonin and cortisol blood samples were obtained from the arterial line. Nurses used a small flashlight and did not wake up the patients or turn on the light when drawing the blood. Eight sound level metres were positioned near the patients’ heads for one week of continuous measurement. The average noise level metre was 62.50 dB(A) during the day and 58.85 dB(A) during the night.

### Measurement of serum melatonin and plasma cortisol levels

Since direct measurement of circadian rhythm is not generally feasible, the secretion of melatonin and cortisol is commonly used as a surrogate for circadian rhythmicity [6]. Normally, melatonin exhibits a peak during the rest period, while cortisol demonstrates a peak during the inactivity to activity transition period [18]. Both were measured three times a day on the first three days after admission to the ICU. Samples were drawn at the specified time according to human physiology. Melatonin samples were drawn at 3:00, 8:00 and 16:00, while cortisol samples were drawn at 0:00, 8:00 and 16:00. We assessed cortisol levels in a subgroup of patients who had not used corticosteroids during the experimental period. The melatonin blood sample was immediately centrifuged and stored at -80 °C for later analysis. Serum melatonin was measured by a melatonin ELISA® kit (Abcam, USA). Plasma cortisol was tested at the central laboratory in Zhongshan Hospital, Fudan University.

### Sleep quality

Sleep quality was assessed using the RCSQ [19], which was translated into Chinese by Li-Xia Chen [20]. The RCSQ has been extensively used to assess sleep quality in ICU patients. It consists of five items, and each item is answered on a 100 mm visual analogue scale. The total score was the arithmetic average of the scores obtained from five items. Scores ranging from 0–25 are considered to indicate poor sleep, and scores ranging from 76–100 are considered to indicate good sleep. Sleep quality was evaluated every day at 8:00–10:00 by trained nurses, except for patients who were on mechanical ventilation.

### Delirium assessment

We assessed delirium twice a day (from 8:00–10:00 and 20:00–22:00) from day 1 after ICU admission and continued for two weeks or until ICU discharged. Delirium was diagnosed by means of the Confusion Assessment Method for the Intensive Care Unit (CAM-ICU) [2, 21]. We defined delirium as at least one positive screening during the ICU stay. Prior to the study, all assessors were trained by a psychiatrist to use the CAM-ICU. Two assessors used the RASS and CAM-ICU to screen patients. When they disagreed with the result, one psychiatrist made the final decision.

Delirium assessment was performed in two steps. First, level of consciousness was assessed using the RASS [22]. This scale has 10 levels ranging from -5 (unarousable) to + 4 (combative). If the patient was in deep sedation (RASS < -3), assessment was stopped and repeated later. Second, the CAM-ICU was used to screen for delirium. It takes 2–5 min to complete. The Chinese version of the CAM-ICU was translated by Heng-Jing Zou [23]. It indicates four features of delirium: (a) an acute onset of changes or fluctuations in mental status; (b) inattention; (c) disorganized thinking; and (d) an altered level of consciousness. The patient is determined to be CAM-ICU positive if the patient manifests both features (a) and (b) plus either (c) or (d) [21].

### Sample-size estimation

To determine the sample size, prior to this study, we conducted preliminary experiments. In accordance with Song et al. [15], we used melatonin at 3:00 on day 1 to measure the sample size. The melatonin levels at 3:00 on day 1 after admission to the ICU were 99.28 ± 57.11 and 48.73 ± 27.44 in the delirium and non-delirium groups, respectively (n = 5 each). The calculation resulted in a minimum sample size of 13 patients per group. Considering a dropout rate of 20%, the sample size was determined to be 32 patients (16 in each group).

### Statistical analysis

All data were analysed with SPSS 22.0 (IBM Corporation) and R 3.6.3 (The R Foundation for Statistical Computing). Continuous data are expressed as the median (25^th^ to 75^th^ percentiles) or mean ± standard deviation, and categorical data are presented as percentages. The Pearson χ2 test was applied to all categorical variables for demographic and medical characteristics data. Normally distributed continuous variables were compared between the delirium and non-delirium groups using the independent sample T test; otherwise, the Mann‒Whitney U test was used. We used CircaCompare [24] to measure the differential rhythmicity of melatonin between delirium and non-delirium patients. The mesor is the average level of values over 24 h [15]. Amplitude corresponds to the distance between the mesor and the peak of the wave [15]. Acrophase is the phase of the maximal value assumed by the curve [15]. Missing values of continuous variables were imputed with the mean, and there were no missing values of categorical variables. P values less than 0.05 were considered statistically significant.

## Result

### Demographic and medical characteristics

A total of 76 patients were included in the study. During the study period, 17 patients developed delirium, resulting in an overall ICU delirium rate of 22.37% (17 of 76). Table 1 shows the demographic and medical characteristics of the patients. There were significant differences in APACHE II score, intubation in ICU, dexmedetomidine use and length of ICU stay between the two groups.

**Table 1:** Characteristics of patients with and without delirium

Characteristics	Total (*n* = 76)	Delirium (*n* = 17)	Non-delirium (*n* = 59)	*P* value
Age (years), mean ± SD	65.08 ± 11.14	66.41 ± 10.52	64.69 ± 11.37	0.565
Sex				0.521
Male, n (%)	56(73.68%)	11(64.71%)	45(76.27%)	
Female, n (%)	20(26.32%)	6(35.29%)	14(23.73%)	
APACHE II	8.80 ± 5.40	11.00(7.50,15.00)	7.00(5.00,11.00)	0.025^*^
Medical history				
Diabetes, n (%)	13(17.11%)	4(23.53%)	9(15.25%)	0.665
Hypertension, n (%)	39(51.32%)	10(58.82%)	29(49.15%)	0.482
History of surgery, n (%)	37(48.68%)	9(52.94%)	28(47.46%)	0.690
History of chemotherapy, n (%)	19(25.00%)	3(17.65%)	16(27.12%)	0.634
Coronary heart disease, n (%)	4(5.26%)	2(11.76%)	2(3.39%)	0.456
Arrhythmology, n (%)	5(6.58%)	1(5.88%)	4(6.78%)	1.000
Valvular disease, n (%)	2(2.63%)	1(5.88%)	1(1.69%)	0.928
Surgery type				0.089
General surgery, n (%)	30 (39.47%)	7 (41.18%)	23 (38.98%)	
Thoracic surgery, n (%)	35 (46.05%)	5 (29.41%)	30 (50.85%)	
Vascular surgery, n (%)	6 (7.89%)	3 (17.65%)	3 (5.08%)	
Orthopaedics, n (%)	2 (2.63%)	1 (5.88%)	1 (1.69%)	
Urology, n (%)	2 (2.63%)	0 (0.00%)	2 (3.39%)	
Gynaecology, n (%)	1 (1.32%)	1 (5.88%)	0 (0.00%)	
Anaesthesia type				0.060
GA	43 (56.58%)	13 (76.47%)	30 (50.85%)	
GA + EA	33 (43.32%)	4 (23.53%)	29 (49.15%)	
Intubation in OR, n (%)	20(26.32%)	7(41.18%)	13(22.03%)	0.205
Intubation in ICU, n (%)	6(7.89%)	4(23.53%)	2(3.39%)	0.028^*^
Time of mechanical ventilation(h)	30.19 ± 27.09	33.88(16.81,71.63)	15.25(9.00,27.65)	0.077
Use of sedatives				
Propofol, n (%)	21(27.63%)	8(47.06%)	13(22.03%)	0.084
Dexmedetomidine, n (%)	14(18.42%)	7(41.18%)	7(11.86%)	0.017^*^
Remifentanil, n (%)	19(25.00%)	7(41.18%)	12(20.34%)	0.153
Fentanyl, n (%)	3(3.95%)	2(11.76%)	1(1.69%)	0.241
Length of ICU stay (days)	3.42 ± 1.90	4.00(3.00,6.50)	2.00(2.00,3.00)	0.002^*^

*APACHE II* Acute Physiology and Chronic Health Evaluation II score, *OR* Operation room, *GA* General anaesthesia, *GA* + *EA* General anaesthesia + Epidural anaesthesia

^*^P value <0.05

### Serum melatonin and plasma cortisol levels

Tables 2 and 3 show the serum melatonin and plasma cortisol levels at the indicated time points during the first three days between the delirium and non-delirium groups. The difference in serum melatonin levels was statistically significant at almost all points except for day 1 at 3:00 and 16:00 and day 2 at 16:00. The plasma cortisol level was measured in 72 (94.74%) patients. There was a significant difference at 16:00 on the first day (*p* = 0.025), and differences at the other time points were not significant (Table 3). Additionally, all variation coefficients of both serum melatonin and plasma cortisol levels showed no significant differences.

**Table 2:** Serum melatonin level(pg/ml)

	N	Total	N	Delirium	N	Non-delirium	*P* value
Day 1							
3:00	73	97.33 ± 48.83	15	97.33(48.97,97.33)	58	97.33(80.72,97.33)	0.123
8:00	76	76.62 ± 80.83	17	41.61(28.18,72.46)	59	62.79(50.20,76.62)	0.048^*^
16:00	76	77.41 ± 91.17	17	42.40(25.54,68.88)	59	61.73(41.52,80.10)	0.084
CV	76	0.40 ± 0.38	17	0.23(0.09,0.97)	59	0.26(0.12,0.55)	0.940
Day 2							
3:00	76	138.72 ± 151.56	17	58.80(36.13,95.52)	59	106.09(68.34,162.12)	0.002^*^
8:00	76	75.39 ± 67.80	17	47.35(29.54,66.47)	59	64.54(45.40,89.27)	0.009^*^
16:00	50	72.55 ± 74.34	15	41.71(27.35,70.15)	35	61.12(29.24,94.63)	0.216
CV	76	0.42 ± 0.31	17	0.29(0.10,0.36)	59	0.41(0.24,0.63)	0.050
Day 3							
3:00	48	132.73 ± 122.13	15	53.52(31.74,115.75)	33	114.93(62.91,179.48)	0.032^*^
8:00	48	76.10 ± 74.62	15	38.88(15.94,70.20)	33	75.66(42.21,119.87)	0.014^*^
16:00	26	68.03 ± 74.82	11	34.54(18.29,68.03)	15	68.03(39.63,93.22)	0.047^*^
CV	48	0.45 ± 0.36	15	0.59(0.15,0.75)	33	0.36(0.14,0.55)	0.225
All CV	76	0.50 ± 0.35	17	0.44(0.20,0.79)	59	0.39(0.24,0.70)	0.704

^*^P value <0.05

**Table 3:** Plasma cortisol level (nmol/L)

	N	Total	N	Delirium	N	Non-delirium	*P* value
Day 1							
0:00	65	566.29 ± 285.80	14	566.29(562.22,567.22)	51	566.29(566.29,566.29)	0.771
8:00	72	547.46 ± 364.21	16	434.50(231.75,606.25)	56	547.46(361.00,695.75)	0.278
16:00	72	479.68 ± 331.88	16	305.00(122.50,386.75)	56	475.00(315.50,636.50)	0.025^*^
CV	72	0.40 ± 0.36	16	0.36(0.27,0.53)	56	0.26(0.17,0.46)	0.122
Day 2							
0:00	72	334.97 ± 156.23	16	298.00(193.25,414.00)	56	332.50(233.00,425.00)	0.416
8:00	72	422.07 ± 187.67	16	381.00(294.50,457.75)	56	392.00(331.25,518.50)	0.401
16:00	48	403.27 ± 187.65	14	362.57 ± 203.72	34	420.03 ± 181.13	0.340
CV	72	0.32 ± 0.23	16	0.31(0.17,0.58)	56	0.26(0.17,0.39)	0.364
Day 3							
0:00	47	311.88 ± 152.67	14	276.84 ± 154.22	33	326.74 ± 151.92	0.311
8:00	47	400.85 ± 135.76	14	347.00(223.25,525.25)	33	389.00(339.50,465.50)	0.377
16:00	25	397.99 ± 177.43	10	399.27 ± 198.82	15	397.13 ± 168.98	0.977
CV	47	0.31 ± 0.24	14	0.31(0.13,0.51)	33	0.22(0.11,0.37)	0.295
All CV	72	0.46 ± 0.27	16	0.46(0.31,0.58)	56	0.44(0.28,0.52)	0.303

^*^P value <0.05

### Circadian rhythm of melatonin and cortisol

**Fig. 1:**
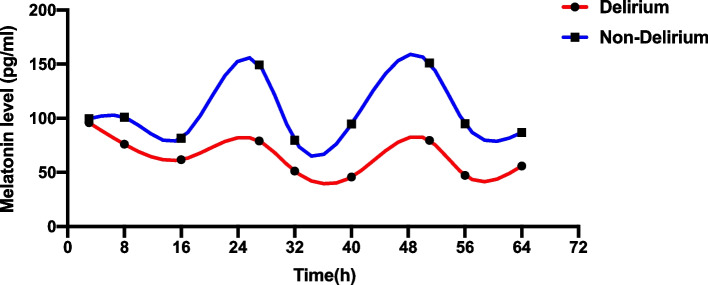
Circadian rhythm of melatonin with and without delirium. Data were shown as mean. Delirium group: mesor:67.60 amplitude:21.78 acrophase:23.33. Non-delirium group: mesor:102.93 amplitude:42.85 acrophase:0.18

**Fig. 2:**
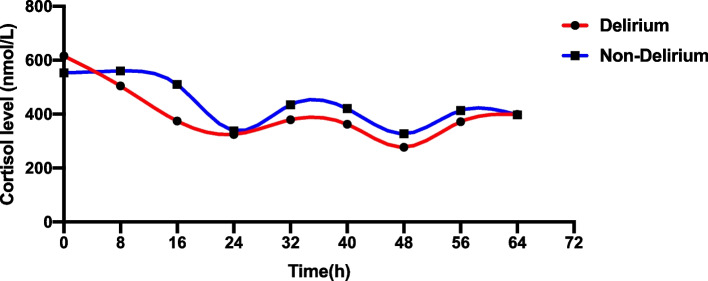
Circadian rhythm of cortisol with and without delirium. Data were shown as mean. Delirium group: mesor:399.52 amplitude:25.36 acrophase:5.64 Non-delirium group: mesor:450.94 amplitude:39.20 acrophase:11.19

The overall presentation of melatonin levels in ICU patients was rhythmic (*p* < 0.001). In the first three days, there was rhythmicity of melatonin levels in the non-delirium group (*p* < 0.001), while there was no rhythmicity of melatonin levels in the delirium group (*p* = 0.064) (Fig. 1). The cyclic presentation of cortisol levels was similar to that of melatonin levels (Fig. 2), and the overall cortisol level had a rhythmic pattern in ICU patients (*p* = 0.044). In the first three days, there was rhythmicity of cortisol levels in the non-delirium group (*p* = 0.026), while there was no rhythmicity of cortisol levels in the delirium group (*p* = 0.454).

### Richard Campbell Sleep Questionnaire scores

RCSQ scores were not significantly different between the two groups except for the awakenings on day 2 (Table 4).

**Table 4:** Richard Campbell Sleep Questionnaire scores

	Total	Delirium	Non-delirium	*P* value
Day 1	*n* = 58	*n* = 10	*n* = 48	
Total	42.98 ± 21.11	37.00 ± 14.64	44.23 ± 22.14	0.329
Sleep depth	43.88 ± 21.13	40.00(20.00,52.50)	40.00(30.00,60.00)	0.511
Sleep latency	43.28 ± 20.89	36.00 ± 14.30	44.79 ± 21.83	0.229
Awakenings	41.38 ± 21.48	30.00(20.00,50.00)	40.00(22.50,57.50)	0.259
Returning to sleep	42.24 ± 21.44	35.00(20.00,50.00)	45.00(22.50,60.00)	0.414
Sleep quality	44.14 ± 22.40	40.00(20.00,52.50)	50.00(30.00,60.00)	0.517
Day 2	*n* = 67	*n* = 12	*n* = 55	
Total	45.58 ± 21.91	35.00 ± 23.27	47.89 ± 21.12	0.064
Sleep depth	45.52 ± 22.38	35.00(20.00,57.50)	50.00(30.00,60.00)	0.191
Sleep latency	45.37 ± 21.83	30.00(20.00,57.50)	50.00(30.00,60.00)	0.070
Awakenings	44.48 ± 21.34	30.00(20.00,50.00)	50.00(30.00,60.00)	0.022^*^
Returning to sleep	45.37 ± 22.45	35.00(20.00,57.50)	50.00(30.00,60.00)	0.137
Sleep quality	47.16 ± 23.41	35.00(20.00,60.00)	50.00(30.00,70.00)	0.170
Day 3	*n* = 43	*n* = 12	*n* = 31	
Total	47.86 ± 21.16	41.33 ± 16.34	50.39 ± 22.48	0.212
Sleep depth	48.37 ± 21.04	42.50 ± 17.12	50.65 ± 22.20	0.260
Sleep latency	47.44 ± 21.83	40.00(30.00,55.00)	50.00(30.00,70.00)	0.289
Awakenings	45.81 ± 20.73	40.00(30.00,47.50)	50.00(30.00,60.00)	0.211
Returning to sleep	47.91 ± 21.55	40.00(30.00,55.00)	50.00(30.00,60.00)	0.221
Sleep quality	49.77 ± 22.41	40.00(32.50,60.00)	50.00(30.00,70.00)	0.369

^*^P value <0.05

## Discussion

This study aimed to explore the relationship between circadian rhythm and ICU delirium. The results showed that the levels of melatonin and cortisol in ICU patients varied according to obvious biological circadian rhythms. However, patients who developed delirium during the ICU stay showed disturbed circadian rhythms of melatonin and cortisol secretion. The subjective RCSQ scores were not different between the delirium and non-delirium patients.

Critically ill patients are particularly susceptible to circadian disruption, and the most obvious manifestation of circadian dysrhythmia in the ICU is delirium [25]. Several existing studies have been designed to explore the relationship between circadian rhythm and delirium in ICU patients. However, previous studies have several limitations. Olofsson et al. [26] analysed melatonin levels over three consecutive days in eight ICU patients and found that impairment of the melatonin rhythm may play a role in the development of sleep disturbances and delirium. However, the limited sample size could affect the rationality of the study conclusion. Hu et al. [27] tested urine aMT6s, the metabolite of melatonin, as a surrogate for serum melatonin in a simulated ICU environment and showed that the difference in nocturnal urine melatonin level of aMT6s was significant. Nevertheless, the results of a recent study confirmed that urinary melatonin secretion aMT6s is not a reliable measure of the melatonin level in critically ill patients [28]. In our study, we used arterial blood samples to test melatonin and cortisol levels, hoping to obtain a more objective reflection of the circadian rhythm of melatonin secretion in ICU patients.

Melatonin plays multiple physiological roles in the regulation of the sleep–wake cycle [29]. Under normal circumstances, melatonin secretion starts at approximately 21:30 and stops at approximately 7:30, peaking at approximately 3:00 [30]. In delirium patients, we observed a significantly lower melatonin level at 8:00 on day 1, at 3:00 and 8:00 on day 2, and at all three time points on day 3 after ICU admission. Previous studies have been controversial, and different studies have yielded different results in comparisons of melatonin levels between delirium and non-delirium patients. In a study by Oxlund et al. [29], serum melatonin levels were measured at 14:00, 22:00 and 3:00 every day for four consecutive days, and the results indicated no significant effect of suppressed melatonin concentration between delirium patients and non-delirium patients. Ángeles-Castellanos et al. [31] collected saliva melatonin samples at 7:00 and 21:00 from the day of admission until hospital discharge, and the results showed that the melatonin rhythm was lost in patients who developed delirium and that the mean melatonin levels were decreased as early as three days before in patients who developed delirium. This study also observed that salivary melatonin levels were significantly lower at night in hospitalized delirium patients. Wu et al. [32] tested morning urine samples on the day of surgery and on days 1, 2 and 7 after surgery and used the 6-sulfatoxymelatonin (6-SMT) /creatinine ratio to provide a more objective estimate of urine 6-SMT concentration. The high 6-SMT group exhibited a ratio increase of more than threefold postoperatively and presented a significantly higher incidence of postoperative cognitive dysfunction in patients. Therefore, ICU delirium was associated with circadian rhythm. However, these studies involved differences in measures and sample collection timing, which would influence the result.

Our study showed that cortisol levels were not significantly different between delirium and non-delirium patients except at 16:00 on day 1. However, other studies have led to opposite conclusions. Sun et al. [33] collected venous blood samples at 2:00, 6:00, 10:00, 14:00, 18:00 and 22:00 for 24 h during the ICU stay, and higher cortisol levels were found in the delirium group than in the non-delirium group at all observed times. Colkesen et al. [34] obtained blood samples at 6:00 the next morning after admission to the CCU and found that the median cortisol level in delirium patients was significantly higher than that in non-delirium patients. We did not find a significant difference in cortisol levels between delirium and non-delirium patients, which might be due to the use of corticosteroids during the operation. Since all the enrolled patients underwent surgical procedures, the operative injection of dexamethasone or methylprednisolone might have affected the secretion of cortisol. Additionally, cortisol secretion was unstable in ICU patients, which could be affected by several factors. Many clinical studies have confirmed that cortisol levels are easily influenced by the inflammatory response [35, 36] and stress [37]. The relationship between cortisol levels and ICU delirium needs to be further explored in future studies.

Notably, we described the rhythm of melatonin and cortisol using a cosine curve, which was a novel parametrization of a nonlinear curve-fitting procedure to the circadian dataset [24]. Our study not only revealed that the melatonin level was significantly different at most time points but also revealed differences in the melatonin secretion rhythm. In the first three days, the melatonin level exhibited biological rhythmicity in the non-delirium group, while no obvious rhythmicity was observed in the delirium group. Although there was no significant difference in the level of cortisol, we observed obvious rhythmicity in the non-delirium group and rhythm disruption in the delirium group. A previous study [15] revealed rhythmicity in both groups, and the mesor of cortisol rhythm was higher in the delirium group. More studies and larger sample sizes are needed to explore the circadian rhythm of ICU patients using cosine curve analysis in the future.

In our study, we used the Richard Campbell Sleep Questionnaire to assess sleep quality, applying the most widely used method in the ICU. Our results indicated no significant difference, and this was in agreement with many previous studies in which the same questionnaire was used [38, 39]. Kamdar et al. [39] analysed ICU patients’ sleep quality using the RCSQ and found no significant differences in any of the five individual RCSQ sleep quality item ratings. This result may be due to the influence of sedation drugs on delirium and tracheal intubation patients, so the subjective experience of patients may not accurately reflect the objective quality of sleep.

However, there were several limitations in this study. First, we monitored only the first three days of melatonin and cortisol levels. The full circadian rhythm in ICU patients was not described. Second, although we measured noise and light in the ICU, we could not avoid sudden changes in light and noise due to emergency situations. Third, we collected blood samples at 0:00/3:00, 8:00 and 16:00 on the first three days after ICU admission to measure the levels of melatonin and cortisol, seeking to reference the time points corresponding to highest and lowest levels in healthy people. Fourth, we did not consider intraoperative hypotensive events in our study. However, the circadian rhythm might be changed in ICU patients, and the preset observation time points of our study might not depict the real highest and lowest points in ICU patients.

## Conclusion

In summary, our study proved that differences in circadian rhythm occur in ICU delirium and non-delirium patients. Delirium patients had a disordered rhythm of melatonin and cortisol, and their overall melatonin and cortisol levels were lower than those in the non-delirium group. Rhythm disturbance may be associated with ICU delirium. The mechanism by which rhythm disturbance affects delirium needs to be further verified in the future. Clinical staff should pay more attention to the importance of maintaining patients’ circadian rhythms in the ICU.

## Data Availability

The datasets used and/or analyzed during the current study are available from the corresponding author on reasonable request.
